# Phylogeography of the Invasive Fruit Fly Species *Bactrocera carambolae* Drew & Hancock (Diptera: Tephritidae) in South America

**DOI:** 10.3390/insects15120949

**Published:** 2024-11-30

**Authors:** Ezequiel de Deus, Joseane Passos, Alies van Sauers-Muller, Cristiane Jesus, Janisete Gomes Silva, Ricardo Adaime

**Affiliations:** 1 Instituto Federal do Amapá, Rodovia BR 210 KM 3, s/n, Macapá 68909-398, Amapá, Brazil; ezequiel.deus@ifap.edu.br; 2 Departamento de Ciências Biológicas e da Saúde, Universidade Federal do Amapá, Programa de Pós-Graduação em Biodiversidade Tropical, Rodovia JK, Km 4, Macapá 68902-280, Amapá, Brazil; 3 Departamento de Biologia, Centro de Estudos Superiores de Coelho Neto, Universidade Estadual do Maranhão, Rua Antônio Guimarães, s/n, Olha D’Aguinha, Coelho Neto 65620-000, Maranhão, Brazil; 4 Fruit Fly Program, Agricultural Experiment Station, Ministry of Agriculture, Animal Husbandry and Fisheries, Paramaribo, Suriname; aliesmuller@yahoo.com; 5 Laboratório de Proteção de Plantas, Embrapa Amapá, Rodovia JK, Km 5, nº 2600, Macapá 68903-419, Amapá, Brazil; cristiane.jesus@embrapa.br; 6 Departamento de Ciências Biológicas, Universidade Estadual de Santa Cruz, Rodovia Jorge Amado, Km 16, Ilhéus 45662-900, Bahia, Brazil

**Keywords:** Brazil, carambola fruit fly, genetic diversity, quarantine pest, Suriname

## Abstract

The carambola fruit fly, *Bactrocera carambolae* Drew & Hancock, is native to Southeast Asia, infests about 150 plant species, and is considered a quarantine pest insect in several regions of the world. *Bactrocera carambolae* has invaded Suriname, French Guyana, and northern Brazil. In Brazil, it was first recorded in 1996 and has been restricted to the states of Amapá and Roraima due to official control efforts of the Ministry of Agriculture and Food Supply (Ministério da Agricultura e Pecuária—MAPA). This is the first study to estimate the genetic structure and diversity of South American populations of *B. carambolae*. A total of 116 individuals from 11 localities in Brazil and 7 localities in Suriname were analyzed. Additional sequences available at GenBank from Indonesia (Lampung) and Thailand (San Pa Tong and Muang District) were also used in the analysis. We sequenced a fragment of the mitochondrial gene cytochrome oxidase subunit I. A total of 35 haplotypes were found. Haplotypes from Indonesia were closest to the haplotypes from South America, separated only by a few mutational steps. This suggests that Indonesia is the likely source for the introduction of *B. carambolae* into South America. The Southeast Asian populations appeared as the most ancestral group in the phylogenetic trees. The high similarity and sharing of several haplotypes among populations within South America indicate a lack of genetic structure. The mismatch distribution and neutrality tests suggest that South American populations have undergone a rapid growth and expansion following a single founder event. The low genetic diversity and the population expansion evidenced by the neutrality tests lend support to the hypothesis of a recent introduction of a single lineage of the carambola fruit fly into South America.

## Simple Summary

This study assessed the genetic diversity of carambola fruit fly populations in Brazil and Suriname, comparing them with Asian populations. The results suggest that Indonesia is the likely source of this species’ introduction into South America, and the low genetic diversity supports the hypothesis of a recent introduction of a single lineage. Additionally, our findings may be useful for invasion risk assessment and for establishing priorities in the control and management of this important pest species.

## 1. Introduction

Fruit flies (Diptera: Tephritidae) are known worldwide as important pests due to their direct economic impact on fruit production as well as the strict quarantine restrictions imposed by importing countries [1]. The Tephritidae comprises more than 5000 species in about 500 genera [2]. In South America, species in the endemic genus *Anastrepha* Schiner and the introduced species *Ceratitis capitata* (Wiedemann) and *Bactrocera carambolae* Drew & Hancock can cause considerable damage to both commercial and non-cultivated fruit species [3,4,5,6].

*Bactrocera* Macquart is one of the largest genera within the Tephritidae with more than 500 described species and is the predominant genus in the Southeast Asia and Pacific regions [7,8]. Some species within this genus are considered highly invasive due to their polyphagous nature, life history strategy, a strong tendency for adult dispersal, and the ease of distribution via transport of immature larval stages inside fruits. These traits together with the globalization of trade and human movement have contributed to their establishment outside their original range [9,10].

The carambola fruit fly, *Bactrocera carambolae*, is native to Indonesia, Malaysia, and Thailand [11]. It has been suggested that the introduction of this species into South America most likely happened in the 1970s due to an increase in the movement of people and agricultural goods. *Bactrocera carambolae* was reported for the first time in South America in 1975 in Paramaribo, Suriname [12]. It was later detected in Guyana (1986), French Guiana (1989), and in Brazil (1996). In Brazil, *B. carambolae* is restricted to the states of Amapá and Roraima under strict official control as it is an important quarantine pest. In the northern region of Brazil, multiple sporadic foci of infestation by *B. carambolae* have been detected and eradicated in the state of Pará, close to the state border with Amapá [5,13,14,15,16,17,18]. In its center of origin, *B. carambolae* has been reported to infest 75 species in 26 plant families [19], whereas in Suriname it infests 20 hosts in 9 families [20]. Recent studies in Brazil have reported 30 plant species in 13 families as hosts for this species, corroborating a decrease in host range in introduced areas compared to where it is native [21,22,23].

*Bactrocera carambolae* belongs to the *Bactrocera dorsalis* complex, which comprises almost 100 taxa that are morphologically highly similar. This species is among the most economically important pests worldwide along with *Bactrocera dorsalis* (Hendel) [24,25,26]. *Bactrocera carambolae* is still a valid taxon as opposed to *Bactrocera papayae* Drew & Hancock, *Bactrocera philippinensis* Drew & Hancock, and *Bactrocera invadens* Drew, Tsuruta, & White. These formerly valid species were synonymized recently with *B. dorsalis* based on studies using integrative taxonomy [27].

Several molecular studies on the pest species of *Bactrocera* have been published in the last decades due to its high impact and significant threat to agricultural resources [8,10,25,26,28,29,30,31,32]. However, despite its importance as a quarantine species, there is a paucity of studies on *B. carambolae* using molecular markers. The only two previous studies using molecular markers are a multilocus phylogeny of pest species in the *Bactrocera dorsalis* complex [8] and a population study on *B. carambolae* and *B. dorsalis* populations using microsatellites [31]. In the latter study, Aketarawong et al. [31] analyzed populations of *B. carambolae* from Southeast Asia (i.e., Indonesia, Malaysia, and Thailand) and South America (one collection from Suriname). The results revealed that the Suriname population is genetically distinct from the Southeast Asian populations and suggest that West Sumatra and Java are the likely sources for the Suriname population.

The mitochondrial gene cytochrome oxidase I (COI) has been useful for phylogenetic and phylogeographic studies on tephritid species in the genera *Anastrepha*, *Bactrocera*, and *Ceratitis* [29,30,33,34,35,36,37,38]. As COI sequences have been employed in several studies on tephritids, abundant data are available for comparative studies [30,39]. Despite being an important quarantine species in Brazil, no genetic studies on populations of *B. carambolae* within the country had been carried out. Therefore, this study aimed at assessing the genetic diversity of populations of *B. carambolae* from Brazil and Suriname and comparing these populations to Asian populations using sequences of the mitochondrial gene cytochrome oxidase I.

## 2. Materials and Methods

### 2.1. Sampling

**Figure 1:**
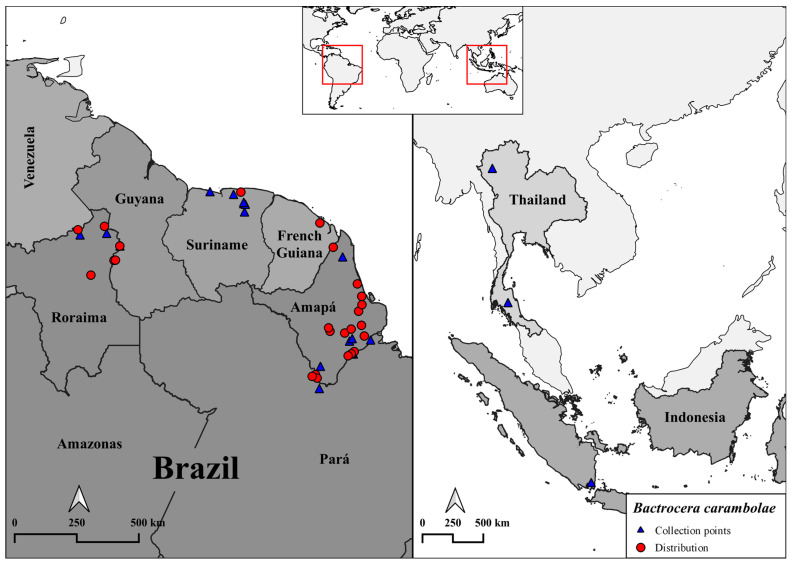
Current distribution of *Bactrocera carambolae* in South America (red dots). Geographic locations of the 18 collection sites (blue triangles).

A total of 116 specimens of *B. carambolae* were either reared from fruit or collected in McPhail traps in 11 localities in Brazil in the states of Amapá, Pará, and Roraima, as well as in 6 localities in Suriname in the districts of Coronie, Saramacca, Brokopondo, and Wanica (Table 1 and Figure 1). Additional sequences available at GenBank previously published by Boykin et al. [8] from Paramaribo (KC446078P, KC446077, KC446076, KC446075, KC446070, KC446069, KC446068, KC446067, KC446066, KC446065, KC446064), Lampung (KC446150, KC446149, KC446147, KC446146), San Pa Tong (KC446152), and Muang District (KC446104, KC446100, KC446099, KC446098) were also used in the analysis (Table 1). Voucher specimens were stored in 100% ethanol at −20 °C at the Laboratório de Entomologia da Embrapa Amapá, Macapá, Amapá, Brazil.

### 2.2. DNA Extraction, PCR Amplification, and Sequencing

Genomic DNA was extracted from three legs of each specimen included in this study using the DNeasy™ Tissue Kit (Qiagen Inc., Valencia, CA) following the manufacturer’s instructions. For the molecular phylogenetic analysis, a fragment of the mitochondrial gene cytochrome oxidase subunit I gene (COI) was amplified using primers LCO1490/HCO2198 [40]. DNA amplification was carried out in 25 μL volume reactions: 12.7 µL ultra-pure water, 2.5 µL 10X buffer, 3.0 µL 25 mM MgCl2, 2.5 µL 100 mMdNTP, 1 µL of each primer (20 mM), 20 ng of DNA, and 2U of Taq DNA polymerase (Promega). PCR conditions were as follows: an initial step at 94 °C for 3 min, followed by 35 cycles (denaturation at 94 °C for 1 min, annealing at 50 °C for 1 min, and extension at 72 °C for 2 min), and a final extension step at 72 °C for 10 min using an Eppendorf^®^ Mastercycler thermocycler. PCR products were purified using exonuclease I of *Escherichia coli* (EXOI) and shrimp alkaline phosphatase (SAP) at the Laboratório de Marcadores Moleculares, Centro de Biotecnologia e Genética, Universidade Estadual de Santa Cruz. PCR products were sequenced asymmetrically using 3′ BigDye-labeled dideoxynucleotide triphosphates run on an ABI 3730 of Life Technologies Applied Biosystems at the Centro de Estudos do Genoma Humano, Universidade de São Paulo. The sequences were submitted to GenBank under accession numbers KX712148-KX712224.

### 2.3. Data Analysis

Sequences were edited and aligned using BIOEDIT version 7.0.5.2 [41]. All sequences were translated and the variable and informative sites were verified using Mega 6.0 [42]. Haplotype and nucleotide diversity (H and π) and haplotype number were estimated using DNAsp 5.0 [43]. The nucleotide substitution model for the Bayesian analyses was determined using the Akaike information criterion implemented in the program jModelTest 2.1.3 [44]. The best fit substitution model for maximum-likelihood analysis was estimated using MEGA 6. DNA sequences of *Bactrocera musae* and *Bactrocera tryoni* used as outgroups were obtained from GenBank (accession numbers KC446039 and KC446030). Phylogenetic trees were generated by Bayesian and Maximum Likelihood analyses using MrBayes 3.2.3 [45] and MEGA 6.0, respectively.

The Bayesian analyses were performed with two simultaneous and independent runs of the Markov Chain Monte Carlo (MCMC) and for 100 million generations with trees sampled every 10,000 generations. Convergence of the two MCMC independent runs and burn-in were accessed in Tracer 1.6 [46]. The first 20% of the trees was discarded as burn-in. Trees were edited in FigTree v1.4.2 [47]. To infer the relationships among haplotypes and their geographic distribution, we used the median-joining network approach [48] Network 5.0 (https://fluxus-engineering.com, accessed on 8 July 2016). The level of genetic structure among populations was estimated by F-statistics and analysis of molecular variance (AMOVA) at two and three hierarchical levels using Arlequin 3.3.11 [49].

The demographic parameters Tajima’s D [50], Fs’s Fu [51,52], and mismatch distribution were estimated to test for the occurrence of demographic population expansions for the sampled population using DNAsp 5.0 [43].

## 3. Results

**Figure 2:**
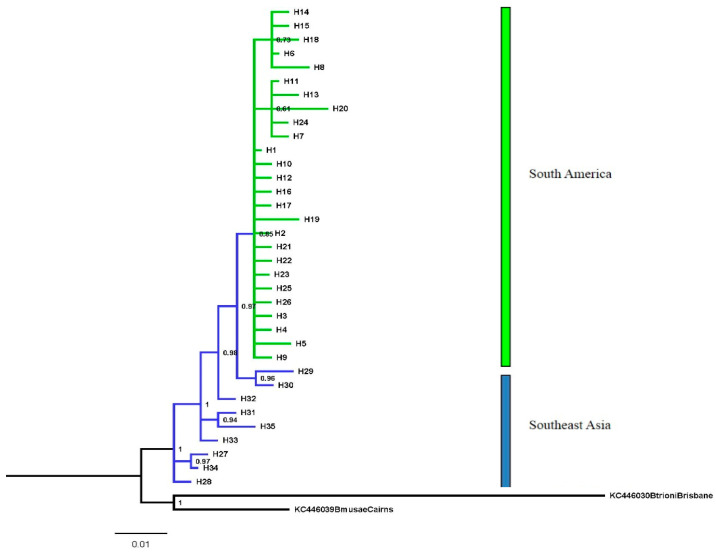
Bayesian phylogeny of *Bactrocera carambolae* haplotypes using the HKY + I model for the mitochondrial gene COI. DNA sequences of *Bactrocera musae* and *Bactrocera tryoni* used as outgroups were obtained from GenBank (accession numbers KC446039 and KC446030). Numbers above internal nodes show posterior probabilities.

**Figure 3:**
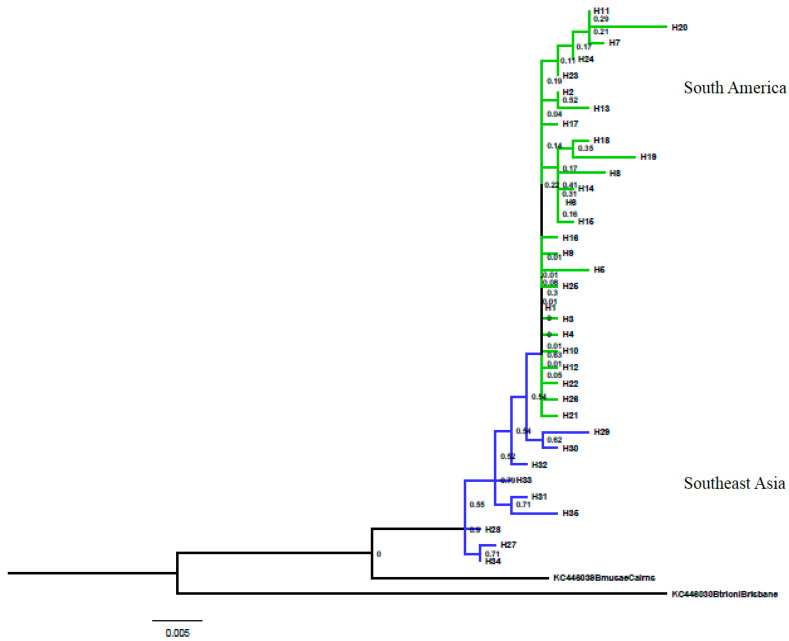
Maximum Likelihood tree using the T92 model with 1000 bootstrap replicates. Numbers above internal nodes show bootstrap support > 50%.

The sequences analyzed were 642 pb long. The fragment sequenced contained 48 variable sites, 16 informative sites, and no indels. A total of 35 haplotypes were identified, and the haplotype and nucleotide diversities for the entire dataset were 0.6607 (±0.046) and 0.00254 (±0.00037), respectively. These indices were estimated for each population and showed moderate values (Hd > 0.5 and π > 0.005). The population of Pacaraima (RR) showed the highest value of haplotype diversity (0.911 ± 0.077) with seven haplotypes (Table 2). The model of molecular evolution selected for the Bayesian analysis was the HKY + I model and that for the Maximum Likelihood analysis was the T92 + G model. Both trees had a similar topology with a monophyletic group, which showed a close relationship between the populations from South America (Brazil and Suriname) and South Sumatra, Lampung, Indonesia (PP = 0.97). The populations from Southeast Asia (Thailand and Indonesia) were clustered with each other (PP = 0.99) (Figure 2 and Figure 3).

Haplotypes H29 and H30 from Indonesia were closest to haplotypes from South America and were separated by only a few mutational steps. This result suggests that Indonesia is the likely source for the introduction of *B. carambolae* into South America. The South American group was divided into two subgroups. The first subgroup (a) comprised predominantly unique haplotypes H13, H20, H24, and H7 and also haplotype H11 found in Pacaraima (RR), Ilha de Santana, Mazagão, and Macapá (AP), and also Jenny (SUR). The second subgroup (b) is represented by the unique haplotypes H14, H15, H18, and H8 and also haplotype H6 shared by the populations of Macapá, Ferreira Gomes (AP), Pacaraima, and Normandia (RR) and also Wanica (SUR). A total of 16 unique haplotypes was observed for the northern region of Brazil and three unique haplotypes for Suriname (Table 1). The Southeast Asian populations appeared as the most ancestral clade in the phylogenetic trees. Group 1 comprising Brazil and Suriname showed a lower haplotype diversity and sequence variation as compared with Group 2, which included Indonesia and Thailand (Table 2).

### Population Structure

**Figure 4:**
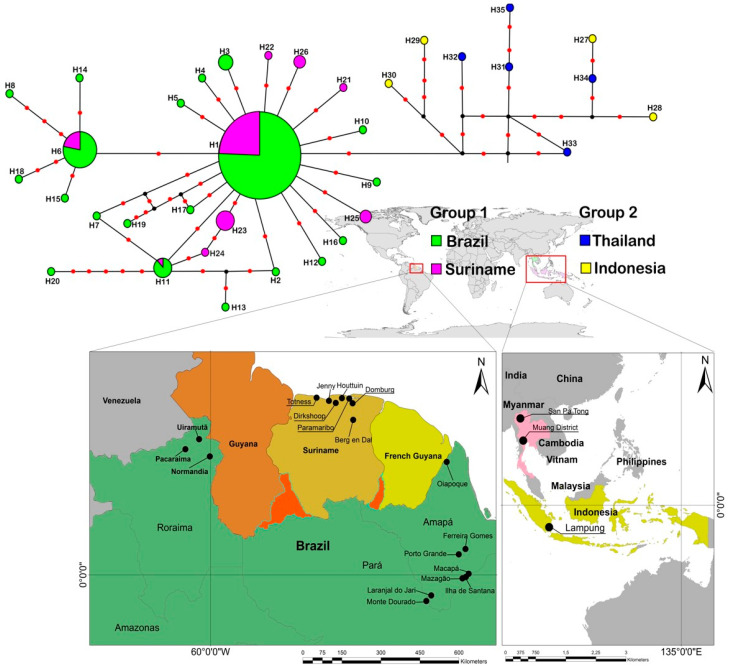
Haplotype network of COI sequences of *Bactrocera carambolae*. Sampled haplotypes are indicated by colored circles, small solid red circles represent mutational steps, and small solid black circles represent median vectors that can be an extinct or unsampled haplotype. Haplotypes are colored according to their geographic origin. Group 1—South America with haplotypes from populations from Brazil and Suriname; Group 2—Southeast Asia with haplotypes from Indonesia and Thailand.

The haplotype network shows the relationships among haplotypes. Each circle represents a haplotype, and circle sizes are proportional to haplotype frequency. Overall, the network is star-shaped with a widely distributed haplotype of high frequency (H1), which lies centrally on the network and is connected to several haplotypes (Figure 4). The topology of the haplotype network revealed two groups, the first with South American populations and the second with Southeastern Asian populations. The high similarity and sharing of several haplotypes among populations within the South American group A indicated a lack of genetic structure. The central haplotype in the network (H1) was the most abundant in the South American group and was found in all localities within this group.

The AMOVA and ɸ_ST_ of populations in this study revealed a lack of genetic structure among the sampled populations in South America. The AMOVA was performed to test two hierarchical levels, considering all populations as a single group in which low levels of genetic structure were observed (ɸ_ST_ = 0.3107 *p* < 0.001). Most of the genetic variation was found within populations (85.44%) (Table 3).

The AMOVA was then performed to test three hierarchical levels, considering South America and Southeast Asia, in which genetic structure was observed between groups (ɸ_ST_ = 0.65294 *p* < 0.001). The highest genetic variation was found between groups (65.29%) indicating differences between populations of Brazil and Southeast Asia (Table 4).

We tested the South American populations for departure from neutrality to evaluate evidence of population expansion using demographic analyses and mismatch distribution of these haplotypes. Overall, the neutrality tests were significant for the populations of South America (*p* < 0.001), Tajima’s D = −2.49844, R2 = 0.08806, and Fu’s Fs = −39.0339. The neutrality tests for Southeast Asian populations were not significant, Tajima’s D = −0.78745, R2 = 0.1098, and Fu’s Fs = −4.518, indicating that those are stable populations.

**Figure 5:**
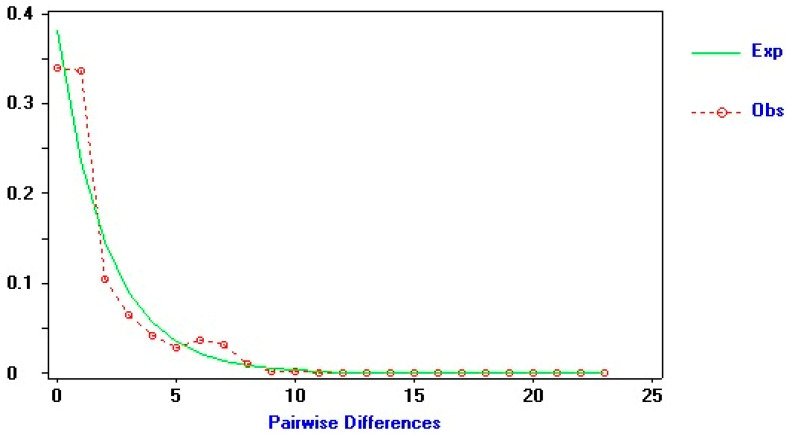
Results of mismatch distributions of the South American populations of *Bactrocera carambolae* analyzed in this study. The continuous line represents the expected frequency, and the observed frequency is represented by a dotted line.

The mismatch distribution of *B. carambolae* haplotypes from South America is unimodal, which, together with the few mutational steps observed among haplotypes in the pairwise comparisons, indicates demographic populations have undergone a recent bottleneck followed by rapid growth and expansion (Figure 5).

## 4. Discussion

This is the first molecular genetic study with an extensive sampling of *B. carambolae* in South America. We had collections from eleven localities in Brazil in the states of Amapá, Pará, and Roraima and from six localities in Suriname in the districts of Coronie, Saramacca, Brokopondo, and Wanica. The studied populations cover most of the geographical distribution of *B. carambolae* in South America. This study also included sequences from individuals collected in the native range of this species deposited at GenBank, which allows for the assessment of dispersal patterns and the source of introduced populations of *B. carambolae*.

Our results revealed the presence of two distinct groups, one comprising populations from South America (Brazil and Suriname) and another group comprising populations from Southeast Asia (Thailand and Indonesia), the latter appearing as the most ancestral group in the phylogenetic trees. The topology of the haplotype network revealed two groups, the first with South American populations and another with Southeast Asian populations. Brazil and Suriname populations showed a lower haplotype diversity and sequence variation as compared with populations from Southeast Asia. The lower genetic variation, the higher similarity, and sharing of several haplotypes among populations observed in the South American populations are consistent with a pattern of colonization following introduction from a source population. Since the introduction into South America is recent, it is likely that the reduction in population size following a genetic bottleneck associated with a population founding event resulted in the loss of alleles and consequent reduction in genetic variation. All non-native invasive species introduced by human-mediated activity have experienced population founding events. Theory predicts that such founding events often result in the establishment of only a fraction of the genetic variation present in the original source population [53]. According to Lynch [54], since genetic drift is stronger in small populations, a reduction in effective population size can lead to a loss of diversity and shorten the time for the fixation of mutations. Therefore, ancestral populations generally show higher levels of gene variation when compared to more recently established populations, which have low diversity and few haplotypes [30,55].

This scenario seems likely since drift and strong selection are likely to drive losses of genetic variation along the first decades of population establishment and growth. Once established, larger populations will undergo reduced drift and can become more interconnected, integrating multiple introductions and showing a rise in genetic variation relative to native source populations. Evolutionary changes that take place after introduction may simply reflect regional differences (i.e., local adaptation, drift, and evolutionary history) between the source population and the introduced population [53]. Our results agree with those of Aketarawong et al. [31] that verified higher genetic variation in the populations within the native range of *B. carambolae* in Southeast Asia than the variation measured in the introduced population in South America (Suriname). Such a scenario has already been described for another tephritid pest species, the Mediterranean fruit fly, *C. capitata*, that is native to Sub-Saharan Africa and is currently the most widespread invasive fruit fly worldwide [33,56,57,58]. A similar pattern of colonization with genetic drift and local adaptation shaping genetic variation has also been reported for *B. dorsalis*, an important pest species native to tropical Asia and which has colonized Hawaii, Myanmar, and Bangladesh, as well as several African countries [27,32,59].

South American populations showed high similarity and shared several haplotypes, which indicate lack of genetic structure. The AMOVA and ɸ_ST_ of populations in this study also revealed a lack of genetic structure among populations in South America and differences between populations of Brazil and Southeastern Asia. The Fst values between groups indicate a lack of genetic structure in populations from Brazil and Suriname, which is generally observed in populations of invasive species in recently colonized areas. Haplotypes H29 and H30 from Indonesia were closest to haplotypes from South America separated by few mutational steps, suggesting that Indonesia is the likely source for the introduction of *B. carambolae* into South America. Our data corroborate the results of the study by Aketarawong et al. [31], which examined populations of *B. carambolae* from Southeast Asia (i.e., Indonesia, Malaysia, and Thailand) and South America (i.e., Suriname) using microsatellites. In that study, the authors indicate that Jakarta and Pekanbaru in Indonesia are possible sources for the Paramaribo, Suriname population. Our results also corroborate the phylogenetic analysis by Boykin et al. [8], in which the only *B. carambolae* population from its invasive range in northern South America emerged as a well-supported distinct subclade “Suriname subclade” within the more diverse clade comprising Southeastern Asian populations (Indonesia, Thailand, and Malaysia). The Suriname population, together with Asian populations, formed a monophyletic clade distinct from other species in the *B. dorsalis* complex. *Bactrocera carambolae* invaded South America via Paramaribo, Suriname, and in 1975 several specimens were reared from curacao apple (*Syzigium samarangense*) but remained unidentified. In 1981, more specimens were reared from the same host and identified as *Dacus dorsalis*. In 1986, new specimens reared from guava (*Psidium guajava*) and sapodilla (*Manilkara zapota*) were identified by A. L. Norrbom together with the specimens from the 1975 fruit collections as *Dacus dorsalis*. Drew and Hancock later examined the Surinamese specimens and proposed that they should be named *Bactrocera carambolae*. As a large part of the Surinamese population originated from Asian countries such as India, Indonesia, and China, the accidental introduction probably occurred by trade or tourists from Indonesia to Suriname [12]. In 1986, the species was reported in French Guyana (about 200 km from Paramaribo). In 1993, *B. carambolae* was found in Orealla, Guyana (about 220 km from Paramaribo), and in 1996 it was found in Oiapoque, Brazil at the border with French Guyana (approximately 500 km from Paramaribo) [13,16,32].

The low genetic diversity and population expansion evidenced by the neutrality test support a recent introduction of the carambola fruit fly into South America followed by rapid growth and expansion. Moreover, the haplotype network shows that the sampled populations are closely related since there are few mutational steps. Similar results were reported for other *Bactrocera* species (cf. [29,60]). Wu et al. [30] compared populations of *B. cucurbitae* from China with those from Southeast Asia and detected a remarkable difference regarding haplotype and nucleotide diversity. Southeast Asian populations showed high levels of genetic variation, whereas Chinese samples showed low levels of diversity. Thus, the authors suggested both that the Southeastern Asian samples can be more similar to the ancestral population and that *B. cucurbitae* has been recently introduced into China.

Our results showed genetic similarities among populations of *B. carambolae* in South America and that Brazilian populations share haplotypes with Surinamese samples. Within the introduced range, free human movement and trading can lead to genetic homogeneity via gene flow, as has been demonstrated for populations within the native range of *B. carambolae* [31]. Thus, a control method (e.g., the sterile insect technique) established for this pest in one area in South America can be used over the introduced range. Moreover, our results can be useful for invasion risk assessment and to establish priorities regarding the control and management of this important pest species.

## Figures and Tables

**Table 1:** Information on *Bactrocera carambolae* specimens used in this study.

Country	District/State	Locality	N	Coordinates	Host	Haplotypes
Suriname	Coronie	Jenny	07	56°05′ W 05°80′ N	*Averrhoa carambola*	H1 (1), H22 (1), H23 (4), H24 (1)
		Totness	06	56°33′ W 05°87′ N	*Averrhoa carambola*	H1 (6)
	Brokopondo	Berg en Dal	06	55°07′ W 05°13′ N	*Averrhoa carambola*	H1 (6)
	Wanica	Houttuin	07	55°10′ W 05°43′ N	*Averrhoa carambola*	H1 (4), H6 (3)
		Domburg	05	55°04′ W 05°41′ N	*Averrhoa carambola*	H1 (2), H6 (1), H11 (1), H21 (1)
	Saramacca	Dirkshoop	07	55°47′ W 05°77′ N	*Averrhoa carambola*	H1(3), H23 (1), H25 (2), H26 (1)
	Paramaribo †	-	11	55°10′ W 05°48′ N	*Averrhoa carambola*	H1 (7)
Brazil	Amapá	Ilha de Santana	10	51°10′ W 00°03′ S	*Eugenia uniflora*	H1 (7), H11 (1), H16 (1), H17 (1)
		Macapá	11	50°47′ W 00°46′ N	*Syzygium malaccense*	H1 (5), H6 (4), H7 (1), H8 (1)
		Oiapoque	9	51°49′ W 03°49′ N	*Psidium guajava*	H1 (7), H3 (2)
		Mazagão	05	51°16′ W 00°05′ S	*Psidium guajava*	H1 (2), H11 (1), H19 (1), H20 (1)
		Porto Grande	04	51°24′ W 00°42’ N	*Psidium guajava*	H1 (3), H18 (1)
		Ferreira Gomes	08	51°14′ W 00°51′ N	*Psidium guajava*	H1 (3), H6 (3), H9 (1), H10 (1)
		Laranjal do Jari	03	52°30′ W 00°50′ S	McPhail traps	H1 (2), H4 (1)
	Roraima	Uiramutã	09	60°10′ W 04°35′ N	*Averrhoa carambola*	H1 (7), H2 (1), H3 (1)
		Pacaraima	10	61°07′ W 04°29′ N	*Averrhoa carambola*	H1 (3), H6 (2), H11 (1), H12 (1), H13 (1), H14 (1), H15 (1)
		Normandia	04	59°37′ W 03°52′ N	McPhail traps	H1 (3), H6 (1)
	Pará	Monte Dourado	05	52°34′ W 01°31′ S	McPhail traps	H1 (4), H5 (1)
Indonesia	South Sumatra †	Lampung	04	105°56′ E 5°40′ S		H27 (1), H28 (1), H29 (1), H30 (1)
Thailand	Chiang Mai †	San Pa Tong	01	98°53′ E 18°37′ N		H31 (1)
	Nakhon Si Thammarat †	Muang District	04	99°53′ E 8°25′ N		H32 (1), H33 (1), H34 (1), H35 (1)

† Sequences from Boykin et al. [8].

**Table 2:** Genetic diversity of *Bactrocera carambolae* populations analyzed in this study.

Population	N	H	Hd (±S.D.)	π (±S.D.)
Jenny-CO	07	4	0.7143 (0.112)	0.00163 (0.00054)
Totness-CO	06	1	-	-
Berg en Dal-BK	06	1	-	-
Houttuin-WA	07	2	0.571 (0.119)	0.00089 (0.00019)
Domburg-WA	05	4	0.900 (0.161)	0.00187 (0.00050)
Dirkshoop-SA	07	4	0.810 (0.130)	0.00163 (0.00040)
Paramaribo-PA	11	2	0.182 (0.144)	0.00028 (0.00022)
Ilha de Santana-AP	10	4	0.533 (0.180)	0.00093 (0.00037)
Macapá-AP	11	4	0.709 (0.099)	0.00227 (0.00079)
Oiapoque-AP	9	2	0.389 (0.164)	0.00061 (0.00026)
Mazagão-AP	05	4	0.900 (0.161)	0.00654 (0.00222)
Porto Grande-AP	04	2	0.500 (0.265)	0.00234 (0.00124)
Ferreira Gomes-AP	08	4	0.786 (0.113)	0.00161 (0.00037)
Laranjal do Jari-AP	03	2	0.667 (0.314)	0.00104 (0.00049)
Uiramutã-RR	09	3	0.417 (0.191)	0.00069 (0.00034)
Pacaraima-RR	10	7	0.911 (0.077)	0.00294 (0.00068)
Normandia-RR	04	2	0.500 (0.265)	0.00078 (0.00041)
Monte Dourado-PA	05	2	0.400 (0.237)	0.00187 (0.00111)
Lampung-SU	04	4	1.000 (0.177)	0.00961 (0.00231)
San Pa Tong-CM	01	1	-	-
Muang District-NT	04	4	1.000 (0.177)	0.00779 (0.00170)
Groups				
South America	127	26	0.611 (0.050)	0.00164 (0.00024)
Asian Southeast	9	9	1.000 (0.052)	0.00865 (0.00110)
Total	136	35	0.6607 (0.046)	0.00254 (0.00037)

CO = Coronie; BK = Brokopondo; WA = Wanica; SA = Saramacca; PA = Paramaribo; AP = Amapá; RR = Roraima; SU = Sumatra; CM = Chiang Mai; NT = Nakhon Si Thammarat; n = number of individuals; h = number of haplotypes; Hd = haplotype diversity; π = nucleotide diversity.

**Table 3:** Analysis of molecular variance for a single group of *B. carambolae* populations.

Source of Variation	Variance Components	Percentage of Variation (%)	ɸ_ST_
Among populations	0.28966	22.43	0.31075
Within populations	0.64247	85.44	

*p* < 0.001.

**Table 4:** Analysis of molecular variance for two groups of *B. carambolae* populations. Group 1—South America and Suriname; Group 2—Southeast Asia.

Source of Variation	Variance Components	Percentage of Variation (%)	ɸ_ST_
Among populations	2.22336	65.29	
Among populations within groups	0.00796	0.42	0.65294
Within populations	0.64247	34.28	

*p* < 0.001.

## Data Availability

The sequences used in this study are available in GenBank under accession numbers KX712148-KX712224.

## References

[1] Aluja M. (1994). Bionomics and Management of *Anastrepha*. Annu. Rev. Entomol..

[2] Zhao Z., Carey J.R., Li Z. (2024). The Global Epidemic of *Bactrocera* Pests: Mixed-Species Invasions and Risk Assessment. Annu. Rev. Entomol..

[3] Zucchi R.A., Vilela E.F., Zucchi R.A., Cantor F. (2001). Mosca-do-mediterrâneo, *Ceratitis capitata* (Diptera: Tephritidae). Histórico e Impacto de Pragas Introduzidas no Brasil.

[4] Vayssières J.F., Cayol J.P., Caplong P., Séguret J., Midgarden D., van Sauers Muller A., Zucchi R., Uramoto K., Malavasi A. (2013). Diversity of fruit fly (Diptera: Tephritidae) species in French Guiana: Their main host plants and associated parasitoids during the period 1994–2003 and prospects for management. Fruits.

[5] Lemos L.N., Adaime R., Jesus-Barros C.R., Deus E.G. (2014). New hosts of *Bactrocera carambolae* (Diptera: Tephritidae) in Brazil. Fla. Entomol..

[6] Qui Y., Paini D.R., Wang C., Fang Y., Li Z. (2015). Global establishment risk of economically important fruit fly species (Tephritidae). PLoS ONE.

[7] Drew R.A.I. (2004). Biogeography and speciation in the Dacini (Diptera: Tephritidae: Dacinae). Bish. Mus. Bull. Entomol..

[8] Boykin L.M., Schutze M.K., Krosch M.N., Chomic A., Chapman T.A., Englezou A., Armstrong K.F., Clarke A.R., Hailstones D., Cameron S.L. (2014). Multi gene phylogenetic analysis of the south-east Asian pest members of the *Bactrocera dorsalis* species complex (Diptera: Tephritidae) does not support current taxonomy. J. Appl. Entomol..

[9] Nentwig W., Nentwig W. (2007). Pathways in Animal Invasions. Biological Invasions.

[10] Khamis F.M., Masiga D.K., Mohamed S.A., Salifu D., De Meyer M., Ekesi S. (2012). Taxonomic identity of the invasive fruit fly pest, *Bactrocera invadens*: Concordance in morphometry and DNA barcoding. PLoS ONE.

[11] White I.M., Elson-Harris M.M. (1992). Fruit Flies of Economic Significance: Their Identification and Bionomics.

[12] van Sauers-Muller A. (1991). An overview of the carambola fruit fly *Bactrocera* species (Diptera: Tephritidae) found recently in Suriname. Fla. Entomol..

[13] Malavasi A., Zucchi R.A., Sugayama R.L., Malavasi A., Zucchi R.A. (2000). Biogeografia. Moscas-Das-Frutas de Importância Econômica no Brasil: Conhecimento Básico e Aplicado.

[14] Godoy M.J.S., Pacheco W.S.P., Portal R.R., Pires Filho J.M., Moraes L.M.M., Silva R.A., Lemos W.P., Zucchi R.A. (2011). Programa Nacional de Erradicação da Mosca-da-carambola. Moscas-Das-Frutas na Amazônia Brasileira: Diversidade, Hospedeiros e Inimigos Naturais.

[15] Brasil Ministério da Agricultura, Pecuária e Abastecimento (2012). Superintendência Federal no Estado do Pará. Portaria No 183.

[16] Malavasi A., Midgarden D., De Meyer M., Peña J.E. (2013). Bactrocera species that pose a threat to Florida: *B. carambolae* and *B. invadens*. Potential Invasive Pests of Agricultural Crops.

[17] Brasil Ministério da Agricultura, Pecuária e Abastecimento (2014). Superintendência Federal no Estado do Pará. Portaria No 55.

[18] Castilho A.P., Pasinato J., Santos J.E.V.D., Nava D.E., Jesus C.R., Adaime R. (2019). Biology of *Bactrocera carambolae* (Diptera: Tephritidae) on four hosts. Rev. Bras. Entomol..

[19] Allwood A.J., Chinajariyawong A., Drew R.A.I., Hamacek E.L., Hancock D.L., Hengsawad C., Jinapin J.C., Jirasurat M., Kong Krong C., Kritsaneepaiboon S. (1999). Host plant records for fruit flies (Diptera:Tephritidae) in South-East Asia. Raffles Bull. Zool..

[20] van Sauers-Muller A. (2005). Host Plants of the Carambola Fruit Fly, *Bactrocera carambolae* Drew & Hancock (Diptera: Tephritidae), in Suriname, South America. Neotrop. Entomol..

[21] Adaime R., Pereira J.D.B., Sousa M.S.M., Jesus C.R., Souza-Filho M.F., Zucchi R.A., Zucchi R.A., Malavasi A., Adaime R., Nava D.E. (2023). Moscas-das-frutas, suas plantas hospedeiras e parasitoides no Estado do Amapá. Moscas das Frutas no Brasil: Conhecimento Básico e Aplicado.

[22] Costa J.V.T.A., Sousa M.S.M., Jesus C.R., Souza-Filho M.F., Costa V.A., Silva B.M.S., Oliveira J.P.M., Adaime R. (2023). New Findings on Carambola Fruit Fly Hosts in South America. Fla. Entomol..

[23] Costa J.V.T.A., Sousa M.S.M., Souza-Filho M.F., Adaime R. (2023). *Chrysophyllum cainito* L. (Sapotaceae): Novo hospedeiro da mosca-da-carambola no Brazil. Agrotrópica.

[24] Clarke A.R., Armstrong K.F., Carmichael A.E., Milne J.R., Raghu S., Roderick G.K., Yeates D.K. (2005). Invasive phytophagous pests arising through a recent tropical evolutionary radiation: The *Bactrocera dorsalis* complex of fruit flies. Annu. Rev. Entomol..

[25] Schutze M.K., Aketarawong N., Amornsak W., Armstrong K.F., Augustinos A.A., Barr N., Bo W., Bourtzis K., Boykin L.M., Caceres C. (2015). Synonymization of key pest species within the *Bactrocera dorsalis* species complex (Diptera: Tephritidae): Taxonomic changes based on a review of 20 years of integrative morphological, molecular, cytogenetic, behavioural and chemoecological data. Syst. Entomol..

[26] Taddei A., Reisenzein H., Mouttet R., Lethmayer C., Egartner A., Gottsberger R.A., Blümel B., Heiss C., Pohn C., Reynaud P. (2023). Morphological and Molecular identification protocols for *Bactrocera dorsalis*: A joint validation study. PhytoFrontiers™.

[27] Schutze M., Mahmood K., Pavasovic A., Bo W., Newman J., Clark A.R., Krosch M.N., Cameron S.L. (2014). One and the same: Integrative taxonomic evidence that *Bactrocera invadens* (Diptera: Tephritidae) is the same species as the Oriental fruit fly *Bactrocera dorsalis*. Syst. Entomol..

[28] Wan X., Nardi F., Zhang B., Liu Y. (2011). The Oriental Fruit Fly, *Bactrocera dorsalis*, in China: Origin and Gradual Inland Range Expansion Associated with Population Growth. PLoS ONE.

[29] Prabhakar C.S., Mehta P.K., Sood P., Singh S.K., Sharma P., Sharma P.N. (2012). Population genetic structure of the melon fly, *Bactrocera cucurbitae* (Coquillett) (Diptera: Tephritidae) based on mitochondrial cytochrome oxidase (COI) gene sequences. Genetica.

[30] Wu Y., McPheron B.A., Wu J.J., Li Z.H. (2012). Genetic relationship of the melon fly, *Bactrocera curcubitae* (Diptera: Tephritidae) inferred from mitochondrial DNA. Insect Sci..

[31] Aketarawong N., Isasawin S., Sojikul P., Thanaphum S. (2015). Gene flow and genetic structure of *Bactrocera carambolae* (Diptera, Tephritidae) among geographical differences and sister species, *B. dorsalis*, inferred from microsatellite DNA data. Zookeys.

[32] Schutze M.K., Bourtzis K., Cameron S.L., Clarke A.R., De Meyer M., Hee A.K.W., Hendrichs J., Krosch M.N., Mwatawala M. (2017). Integrative taxonomy versus taxonomic authority without peer review: The case of the Oriental fruit fly, *Bactrocera dorsalis* (Tephritidae): Integrative taxonomy versus authority. Syst. Entomol..

[33] Meixner M.D., McPheron B.A., Silva J.G., Gasparich G.E., Sheppard W.S. (2002). The Mediterranean fruit fly in California: Evidence for multiple introductions and persistent populations based on microsatellite and mitochondrial DNA variability. Mol. Ecol..

[34] Smith P.T., Kambhampati S., Armstrong K.A. (2003). Phylogenetic relationships among *Bactrocera species* (Diptera: Tephritidae) inferred from mitochondrial DNA sequences. Mol. Phylogenet. Evol..

[35] Li Y., Wu Y., Chen H., Wu J., Li Z. (2012). Population structure and colonization of *Bactrocera dorsalis* (Diptera: Tephritidae) in China, inferred from mtDNA COI sequences. J. Appl. Entomol..

[36] Ruiz-Arce R., Barr N.B., Owen C.L., Thomas D.B., McPheron B.A. (2012). Phylogeography of *Anastrepha obliqua* inferred with mtDNA sequencing. J. Econ. Entomol..

[37] Barr N.B., Ledezma L.A., Leblanc L., San Jose M., Rubinoff D., Geib S.M., Fujita B., Bartels D.W., Garza D., Kerr P. (2014). Genetic diversity of *Bactrocera dorsalis* (Diptera: Tephritidae) on the Hawaiian islands: Implications for an introduction pathway into California. J. Econ. Entomol..

[38] Ruiz-Arce R., Owen C.L., Thomas D.B., Barr N.B., McPheron B.A. (2015). Phylogeographic structure in *Anastrepha ludens* (Diptera: Tephritidae) populations inferred with mtDNA sequencing. J. Econ. Entomol..

[39] Smith-Caldas M.R.B., McPheron B.A., Silva J.G., Zucchi R.A. (2001). Phylogenetic relationships among species of the *fraterculus* group (*Anastrepha*: Diptera: Tephritidae) inferred from DNA sequence of the mitochondrial cytocrome oxidase I. Neotrop. Entomol..

[40] Folmer O., Black M., Hoeh W., Lutz R., Vrijenhoek R. (1994). DNA primers for amplification of mitochondrial cytochrome c oxidase subunit I from diverse Metazoan Invertebrate. Mol. Mar. Biol. Biotechnol..

[41] Hall T.A. (1999). Bioedit: A user-friendly biological sequence alignment editor and analysis program for windows 95/98/NT. Nucleic Acids Symp. Ser..

[42] Tamura K., Stecher G., Peterson D., Filipski A., Kumar S. (2013). MEGA6: Molecular Evolutionary Genetics Analysis version 6.0. Mol. Biol. Evol..

[43] Librado P., Rozas J. (2009). DnaSP v5: A software for comprehensive analysis of DNA polymorphism data. Bioinformatics.

[44] Darriba D., Taboada G.L., Doallo R., Posada D. (2012). jModelTest 2: More models, new heuristics and parallel computing. Nat. Methods.

[45] Ronquist F., Huelsenbeck J.P. (2003). MrBayes 3: Bayesian phylogenetic inference under mixed models. Bioinformatics.

[46] Rambaut A., Suchard M.A., Xie D., Drummond A.J. (2014). MCMC Trace Analysis Tool, v1.6.0.

[47] Rambaut A. (2009). Tree Figure Drawing Tool, Version 1.3.1.

[48] Bandelt H.J., Forster P., Röhl A. (1999). Median-joining networks for inferring intraspecific phylogenies. Mol. Biol. Evol..

[49] Excoffier L., Laval G., Schneider S. (2005). Arlequin v3.11: An integrated software package for population genetics data analysis. Evol. Bioinform. Online.

[50] Tajima F. (1989). Statistical methods for testing the neutral mutation hypothesis by DNA polymorphism. Genetics.

[51] Fu Y.X. (1997). Statistical tests of neutrality of mutations against population growth, hitchhiking and background selection. Genetics.

[52] Ramos-Osins S.E., Rozas J. (2002). Statistical Properties of New Neutrality Testes Against Population Growth. Mol. Biol. Evol..

[53] Dlugosch K.M., Parker M. (2008). Founding events in species invasions: Genetic variation, adaptive evolution, and role of multiple introductions. Mol. Ecol..

[54] Lynch M. (2007). The Origins of Genome Architecture.

[55] Passos J.F., Nascimento D.B., Menezes R.S.T., Adaime R., Araujo E.L., Lima K.M., Roberto Zucchi R.A., Ronchi Teles B., Nascimento R.R., Ruiz-Arce R. (2018). Genetic structure and diversity in Brazilian populations of *Anastrepha obliqua* (Diptera: Tephritidae). PLoS ONE.

[56] Gasparich G.E., Silva J.G., Han J.Y., McPheron B.A., Steck G.J., Sheppard W.S. (1997). Population genetic structure of Mediterranean fruit fly (Diptera: Tephritidae) and implications for worldwide colonization patterns. Ann. Entomol. Soc. Am..

[57] Bonizzoni M., Zheng L., Guglielmino C.R. (2001). Microsatellite analysis of medfly bioinfestation in California. Mol. Ecol..

[58] Silva J.G., Meixner M.D., McPheron B.A., Steck G.J., Sheppard W.S. (2003). Recent Mediterranean fruit fly (Diptera: Tephritidae) infestations in Florida—A genetic perspective. J. Econ. Entomol..

[59] Aketarawong N., Bonizzoni M., Thanaphum S., Gomulski L.M., Gasperi G., Malacrida A.R., Guglielmino C.R. (2007). Inferences on the population structure and colonization process of the invasive oriental fruit fly, *Bactrocera dorsalis* (Hendel). Mol. Ecol..

[60] Hu J., Zhang J.L., Nardi F., Zhang R.J. (2008). Population genetic structure of the melon fly, *Bactrocera cucurbitae* (Diptera: Tephritidae), from China and Southeast Asia. Genetica.

